# Supplementary material for: VeChat: correcting errors in long reads using variation graphs
Source: Nat Commun. 2022 Nov 4;13:6657. doi: 10.1038/s41467-022-34381-8 (PMC9636371; doi:10.1038/s41467-022-34381-8)
Supplement: Supplementary file 1 — Supplementary Information [file 41467_2022_34381_MOESM1_ESM.pdf]

VeChat: Correcting errors in noisy long reads using variation graphs Supplementary Information

Xiao Luo[1,2], Xiongbin Kang[1], Alexander Schönhuth[1,2,*]

[1] Genome Data Science, Faculty of Technology, Bielefeld University, Bielefeld, Germany
[2] Centrum Wiskunde & Informatica, Amsterdam, The Netherlands

[*]To whom correspondence should be addressed.

Supplementary Tables and Figures

Method	#Reads	Error rate (%, QV)	Switch error (%)	Haplotype coverage (%)	N50 (bp)
**HG002 (diploid)**					
VeChat	2385346	0.328	11.6	92.2	53620
CONSENT	2578729	0.478	13.6	97.0	54298
Canu	-	-	-	-	-
Daccord	-	-	-	-	-
Racon	-	-	-	-	-
**Human gut microbiome**					
Daccord	3050430	1.323	-	91.8	4073
VeChat	2733544	1.352	-	91.7	3622
CONSENT	3097015	1.958	-	93.9	4672
Canu	1705153	2.351	-	93.3	5399
Racon	-	-	-	-	-

Supplementary Table 1. Error correction benchmarking results for real ONT sequencing data (non-synthetic). For the human genome sample HG002, we stopped Canu after running 22 days on a computer with 160 CPUs and 2TB RAM due to out of disk space (the size of temporary files > 10TB), so no result was reported. Daccord and Racon failed to run for HG002 dataset. Racon failed to run for the human gut microbiome data. Note that Merqury is used to evaluate the error correction performance of these two datasets since the ground truth is unknown. The 'error rate' and 'haplotype coverage' correspond to the 'consensus quality value (QV)' and 'k-mer completeness' reported by Merqury, respectively. The 'switch error' of human gut microbiome data is unavailable.

	Metrics from Merqury			Metrics from QUAST	
Method	Error rate (%, QV)	Switch error (%)	k-mer completeness (%)	Error rate (%)	Haplotype coverage (%)
**E.coli genomes (Ploidy=2,PacBio)**					
VeChat	0.008	0.16	100.0	0.014	100.0
CONSENT	0.041	6.88	99.9	0.194	99.9
Racon	0.102	5.45	98.5	0.276	99.2
Canu	0.137	4.92	99.7	0.308	99.9
Daccord	0.022	5.46	92.4	0.423	99.2
**E.coli genomes (Ploidy=2,ONT)**					
VeChat	0.014	0.16	99.9	0.022	99.9
CONSENT	0.049	6.95	99.9	0.212	99.9
Racon	0.136	6.17	98.1	0.346	99.3
Canu	0.190	5.44	99.7	0.390	100.0
Daccord	0.037	6.07	92.7	0.438	99.2
**Metagenome (Low complexity,PacBio)**					
VeChat	0.015	-	98.4	0.036	96.9
Racon	0.082	-	95.8	0.200	91.7
CONSENT	0.055	-	98.4	0.214	98.4
Canu	0.122	-	98.4	0.259	97.4
Daccord	0.013	-	90.5	0.259	92.8
**Metagenome (High complexity,PacBio)**					
VeChat	0.029	-	96.4	0.088	97.5
CONSENT	0.096	-	98.8	0.274	99.4
Canu	0.163	-	98.6	0.354	99.0
Racon	-	-	-	-	-
Daccord	-	-	-	-	-

Supplementary Table 2. Comparison between reference-free (Merqury) and reference-based (QUAST) evaluations. We performed error correction benchmarking experiments on 4 simulated datasets (pseudo-diploid *E.coli* genome and metagenome datasets as we described in the 'Datasets' subsection), of which the ground truth are known. QV: consensus quality value. Methods are sorted by the values of column 'Error rate' (QUAST).

Method	#Seq	Error rate (%)	Mismatch (%)	Indel (%)	Haplotype coverage (%)	N50 (bp)	NGA50 (bp)	# Misassemblies
**Coverage=10x**								
VeChat	14960	0.311	0.114	0.197	99.0	12945	30952	21
Racon	16308	0.629	0.433	0.196	95.8	12571	30948	114
Canu	16403	0.830	0.535	0.296	98.9	12584	30903	35
Daccord	15896	0.851	0.749	0.102	98.5	12611	31031	17
CONSENT	16802	1.075	0.708	0.367	98.4	12573	30815	228
**Coverage=20x**								
VeChat	31132	0.070	0.028	0.043	99.9	12765	36029	15
CONSENT	33629	0.517	0.423	0.094	99.9	12559	35572	121
Racon	32644	0.565	0.427	0.138	97.9	12537	35503	190
Daccord	32038	0.813	0.754	0.059	99.5	12550	35605	12
Canu	31288	0.863	0.493	0.370	99.8	12652	35581	35
**Coverage=30x**								
VeChat	48085	0.031	0.015	0.016	100.0	12595	38467	13
CONSENT	50462	0.276	0.205	0.071	100.0	12511	38187	105
Racon	48986	0.558	0.427	0.131	98.7	12484	38257	288
Canu	37210	0.612	0.405	0.207	99.9	13675	38360	30
Daccord	48189	0.807	0.752	0.055	99.7	12485	38357	16
**Coverage=40x**								
VeChat	64475	0.022	0.010	0.012	100.0	12570	40264	20
CONSENT	67255	0.219	0.137	0.083	100.0	12497	40077	114
Canu	50236	0.533	0.334	0.199	100.0	13678	39999	30
Racon	65210	0.561	0.425	0.136	99.1	12482	39925	386
Daccord	64294	0.803	0.749	0.054	99.7	12466	39970	17
**Coverage=50x**								
VeChat	80984	0.017	0.008	0.009	100.0	12537	41609	34
CONSENT	84185	0.211	0.112	0.099	100.0	12477	41284	124
Canu	48663	0.459	0.295	0.164	100.0	14427	41217	28
Daccord	80529	0.804	0.751	0.053	99.8	12428	41311	25

Supplementary Table 3. Error correction benchmarking results for simulated PacBio CLR reads of various sequencing coverages. The average sequencing coverage per haplotype is set as 10x, 20x, 30x, 40x, and 50x, respectively. The ploidy is three and sequencing error rate is 10%.

Method	#Reads	Error rate (%)	Mismatch (%)	Indel (%)	Haplotype coverage (%)	N50 (bp)
**Simulated PacBio CLR**						
VeChat	26162	0.208	0.132	0.076	99.6	1974
GraphAligner	26159	0.383	0.381	0.002	99.9	2626
Daccord	13020	0.565	0.542	0.023	99.8	2908
CONSENT	25738	0.687	0.022	0.665	99.6	2310
Canu	18038	1.345	0.257	1.088	99.9	2678
Racon	26162	1.487	0.783	0.704	99.1	2194
**Simulated ONT**						
CONSENT	24762	0.078	0.003	0.075	99.8	2569
VeChat	24957	0.403	0.246	0.157	99.6	1999
GraphAligner	24976	0.442	0.438	0.004	100.0	2817
Daccord	25824	0.527	0.384	0.143	99.9	2163
Canu	18271	1.009	0.262	0.746	100.0	2913
Racon	26018	1.057	0.502	0.555	99.2	2179

Supplementary Table 4. Benchmarking results of error correction tools on the simulated *5-strain HIV mixture* (genome size ≈10Kbp) dataset. This dataset is one of the most challenging datasets in viral quasispecies assembly, which consists of five known HIV-1 strains (YU2, NL43, JRCSF, HXB2, 896) and has been used for benchmarking experiments in many related studies, such as [1, 2, 3, 4]. The overall sequencing coverage of the virus data in this table is 5000×. The sequencing coverage of each virus strain varies from 500× to 1500× (average coverage is about 1000×). Note that GraphAligner performs hybrid error correction (i.e., using both short and long reads) and requires a de Bruijn graph as input. Thus, we first simulated Illumina short reads (2x150 bp) with the same sequencing coverage for each strain using ART [**?**], then we constructed a de Bruijn graph from short reads using BCALM2 [**?**] and finally we corrected long read sequencing errors with GraphAligner [**?**]. Technically, the results of GraphAligner are incomparable here since all other methods perform self correction.

Method	#Seq	Error rate (%)	Mismatch (%)	Indel (%)	Haplotype coverage (%)	N50 (bp)	NGA50 (bp)	# Mis-assem-blies
**Error=5%**								
VeChat	48624	0.009	0.007	0.003	100.0	12764	38945	19
CONSENT	50144	0.091	0.073	0.018	100.0	12724	38779	80
Racon	48691	0.507	0.440	0.067	98.2	12724	38257	497
Canu	35914	0.539	0.460	0.079	99.8	13996	38468	116
Daccord	47645	0.838	0.784	0.054	99.7	12705	38389	82
**Error=10%**								
VeChat	48085	0.031	0.015	0.016	100.0	12595	38467	13
CONSENT	50462	0.276	0.205	0.071	100.0	12511	38187	105
Racon	48986	0.558	0.427	0.131	98.7	12484	38257	288
Canu	37210	0.612	0.405	0.207	99.9	13675	38360	30
Daccord	48189	0.807	0.752	0.055	99.7	12485	38357	16
**Error=15%**								
VeChat	46829	0.091	0.049	0.042	99.9	12650	36292	67
Racon	48359	0.618	0.390	0.228	99.7	12539	36241	163
CONSENT	50032	0.680	0.429	0.252	99.9	12532	36054	218
Daccord	47914	0.780	0.716	0.064	99.8	12527	36338	5
Canu	39040	0.843	0.364	0.479	99.9	13333	36045	28

Supplementary Table 5. Error correction benchmarking results for simulated PacBio CLR reads with different sequencing error rates of polyploid genome (ploidy=3). The average sequencing coverage per haplotype is 30x and sequencing error rate = 5%, 10%, 15%.

Method	Assembler	#Seq	Error rate (%)	Mismatch (%)	Indel (%)	Haplotype coverage (%)	N50 (bp)	NGA50 (bp)	# Mis-assem-blies
**Ploidy=2**									
VeChat	HiCanu	23	0.003	0.002	0.000	99.6	1828612	1828612	0
VeChat	Canu	61	0.094	0.092	0.001	91.0	262470	187262	21
-	Canu	78	0.272	0.259	0.013	92.0	205190	144312	22
VeChat	Flye	143	0.505	0.486	0.018	60.3	514429	31156	67
-	Flye	110	0.534	0.510	0.024	58.2	669770	22156	70
**Ploidy=3**									
VeChat	HiCanu	132	0.008	0.006	0.002	98.0	522992	746652	3
VeChat	Canu	68	0.080	0.076	0.004	93.2	785131	268828	25
-	Canu	155	0.586	0.523	0.064	89.4	135872	121655	38
VeChat	Flye	297	0.386	0.352	0.034	70.9	117135	44770	61
-	Flye	201	0.572	0.506	0.066	50.8	95477	8965	77
**Ploidy=4**									
VeChat	HiCanu	218	0.047	0.037	0.009	95.1	205995	216404	7
VeChat	Canu	193	0.172	0.145	0.027	82.9	156620	127972	40
-	Canu	223	0.463	0.409	0.054	81.2	130493	97375	50
VeChat	Flye	272	0.420	0.384	0.036	51.2	83062	18859	75
-	Flye	191	0.700	0.634	0.066	39.1	91739	-	85

Supplementary Table 6. Genome assembly benchmarking results for simulated PacBio CLR reads of various polyploid genomes (ploidy=2,3,4). '#Seq' indicates the number of contigs. The error rate is equal to the sum of mismatch and indel rate. The results are sorted by the error rate in ascending order.

Method	Assembler	#Seq	Error rate (%)	Mismatch (%)	Indel (%)	Haplotype coverage (%)	N50 (bp)	NGA50 (bp)	# Mis-assem-blies
**Ploidy=2**									
VeChat	HiCanu	46	0.010	0.007	0.002	99.3	982281	982281	2
VeChat	Canu	64	0.166	0.160	0.006	82.3	502269	107439	44
-	Canu	75	0.348	0.319	0.029	80.4	233272	96336	31
VeChat	Flye	139	0.473	0.444	0.029	60.5	433584	34284	61
-	Flye	94	0.492	0.458	0.034	58.3	1360777	29914	76
**Ploidy=3**									
VeChat	HiCanu	126	0.020	0.013	0.007	93.7	248802	226750	0
VeChat	Canu	124	0.260	0.232	0.028	78.6	143093	87280	62
-	Canu	141	0.680	0.587	0.093	74.0	127375	72167	65
VeChat	Flye	301	0.382	0.348	0.034	69.2	101356	44053	69
-	Flye	177	0.644	0.556	0.088	50.1	99341	5534	86
**Ploidy=4**									
VeChat	HiCanu	310	0.073	0.059	0.014	84.3	104391	88891	10
VeChat	Canu	185	0.293	0.252	0.042	67.7	105299	65081	72
-	Canu	169	0.525	0.452	0.073	62.9	115900	57885	69
VeChat	Flye	263	0.505	0.454	0.051	49.3	75828	9146	73
-	Flye	176	0.687	0.613	0.074	39.1	103955	-	89

Supplementary Table 7. Genome assembly benchmarking results for simulated Oxford Nanopore reads of various polyploid genomes (ploidy=2,3,4). The average sequencing coverage per haplotype is 30x and sequencing error rate is 10%.

Method	Assembler	#Seq	Error rate (%)	Mismatch (%)	Indel (%)	Haplotype coverage (%)	N50 (bp)	NGA50 (bp)	# Mis-assem-blies
**Low complexity (20 genomes)**									
VeChat	HiCanu	830	0.043	0.037	0.006	84.8	387814	301596	35
VeChat	Canu	500	0.129	0.117	0.012	77.6	929383	191909	102
-	Canu	590	0.332	0.303	0.029	75.2	669888	121618	169
VeChat	metaFlye	435	0.216	0.193	0.023	61.7	944262	97891	214
-	metaFlye	531	0.289	0.255	0.034	57.2	970829	45270	294
**High complexity (100 genomes)**									
VeChat	HiCanu	5150	0.092	0.083	0.008	83.7	224683	204953	341
VeChat	Canu	2858	0.185	0.169	0.016	80.0	346778	135755	626
-	Canu	3334	0.384	0.351	0.033	76.3	226577	95276	672
VeChat	metaFlye	3044	0.249	0.227	0.022	57.4	550617	42845	913
-	metaFlye	3554	0.388	0.342	0.046	52.0	264868	15820	1206

Supplementary Table 8. Genome assembly benchmarking results for simulated PacBio CLR reads of metagenomic datasets with different complexity. The average sequencing coverage of strains is about 30x and the sequencing error rate is 10%.

Method	Assembler	#Seq	Error rate (%)	Mismatch (%)	Indel (%)	Haplotype coverage (%)	N50 (bp)	NGA50 (bp)	# Mis-assem-blies
**Yeast pseudo-diploid genome**									
VeChat	HiCanu	472	0.326	0.263	0.063	80.3	102157	69629	41
VeChat	Canu	218	0.507	0.439	0.068	56.8	366616	24292	47
-	Canu	251	0.543	0.452	0.091	60.1	304452	28992	44
VeChat	Flye	70	0.666	0.590	0.076	50.3	815395	-	70
-	Flye	58	0.734	0.632	0.102	49.9	770183	-	56
**NWC metagenome**									
VeChat	HiCanu	198	0.027	0.020	0.007	68.7	265602	47532	74
VeChat	Canu	665	0.072	0.051	0.021	85.8	42744	73912	370
-	Canu	191	0.129	0.057	0.072	85.8	268047	138616	252
VeChat	metaFlye	280	0.093	0.080	0.013	62.5	152648	27218	221
-	metaFlye	364	0.221	0.121	0.100	63.3	101213	24220	338
**Microbial 10-plex metagenome**									
VeChat	HiCanu	1052	0.115	0.101	0.014	91.1	171298	347013	97
VeChat	Canu	536	0.149	0.133	0.017	88.8	422257	132643	107
-	Canu	485	0.205	0.179	0.026	89.0	411086	135250	103
VeChat	metaFlye	363	0.195	0.174	0.021	71.6	2045015	97075	142
-	metaFlye	291	0.208	0.182	0.026	70.5	1248427	102535	153

Supplementary Table 9. Genome assembly benchmarking results for real sequencing data (mock communities).

Method	CPU time (h)	Peak memory usage (GB)
**Ploidy=2**		
CONSENT	4.8	5.3
Daccord	12.7	12.8
Canu	14.7	5.0
Racon	17.9	4.2
VeChat	23.3	10.8
**Ploidy=3**		
CONSENT	8.7	7.8
Canu	14.6	5.1
Daccord	26.0	14.0
Racon	39.0	7.3
VeChat	47.1	24.1
**Ploidy=4**		
CONSENT	13.1	8.8
Canu	22.6	5.1
Daccord	51.7	17.1
Racon	77.2	10.7
VeChat	81.4	29.8

Supplementary Table 10. Runtime and memory usage for simulated PacBio CLR reads of various polyploid genomes (ploidy=2,3,4). The average sequencing coverage per haplotype is 30x and sequencing error rate is 10%.

Method	CPU time (h)	Peak memory usage (GB)
**Ploidy=2**		
CONSENT	4.9	5.1
Daccord	8.4	12.9
Canu	11.6	5.0
Racon	14.2	5.3
VeChat	8.4	28.3
**Ploidy=3**		
CONSENT	8.5	7.5
Canu	11.9	5.2
Daccord	17.4	14.0
Racon	31.2	7.7
VeChat	46.4	50.5
**Ploidy=4**		
CONSENT	13.4	8.8
Canu	28.0	5.0
Daccord	30.9	17.1
Racon	35.6	11.7
VeChat	91.8	54.4

Supplementary Table 11. Runtime and memory usage for simulated Oxford Nanopore reads of various polyploid genomes (ploidy=2,3,4). The average sequencing coverage per haplotype is 30x and sequencing error rate is 10%.

Method	CPU time (h)	Peak memory usage (GB)
**Low complexity (20 genomes)**		
CONSENT	56.5	15.8
Canu	100.8	10.8
Racon	159.3	44.2
Daccord	162.9	8.9
VeChat	393.5	51.0
**High complexity (100 genomes)**		
CONSENT	375.0	17.6
Canu	880.0	10.8
VeChat	2668.7	78.0
Racon	-	-
Daccord	-	-

Supplementary Table 12. Runtime and memory usage for simulated PacBio CLR reads of metagenomic datasets with different complexity. The average sequencing coverage of strains is 30x and sequencing error rate is 10%.

Method	CPU time (h)	Peak memory usage (GB)
**Yeast pseudo-diploid genome**		
CONSENT	5.2	14.0
Canu	29.1	5.2
Racon	33.2	8.3
Daccord	77.7	16.5
VeChat	51.5	42.4
**NWC metagenome**		
CONSENT	24.4	19.7
Canu	26.2	5.0
Daccord	208.6	15.3
Racon	423.1	25.5
VeChat	780.0	61.6
**Microbial 10-plex metagenome**		
CONSENT	37.4	13.2
Canu	26.2	5.0
Daccord	80.8	11.8
Racon	152.7	29.4
VeChat	285.9	40.8

Supplementary Table 13. Runtime and memory usage for real sequencing data (mock communities).

Method	#Reads	Error rate (%)	Mismatch (%)	Indel (%)	Haplotype coverage (%)	N50 (bp)	NGA50 (bp)	# Mis-assem-blies
**Ploidy=2**								
Cycle 1	32244	0.206	0.032	0.174	100.0	12515	38441	1
Cycle 2	31958	0.014	0.006	0.008	100.0	12556	38515	0
**Ploidy=3**								
Cycle 1	49133	0.286	0.066	0.220	100.0	12515	38434	10
Cycle 2	48085	0.031	0.015	0.016	100.0	12595	38467	13
**Ploidy=4**								
Cycle 1	64539	0.317	0.097	0.220	99.9	12494	38432	47
Cycle 2	62743	0.074	0.047	0.027	99.9	12593	38442	44
**Metagenome of low complexity (20 genomes)**								
Cycle 1	299149	0.203	0.038	0.165	98.8	11816	29558	91
Cycle 2	293466	0.036	0.020	0.015	96.9	11866	29555	104

Supplementary Table 14. Results of cycle 1 and cycle 2 in VeChat. Simulated PacBio CLR datasets of polyploid genomes(ploidy=2,3,4) and metagenome (low complexity) are shown.

(a)

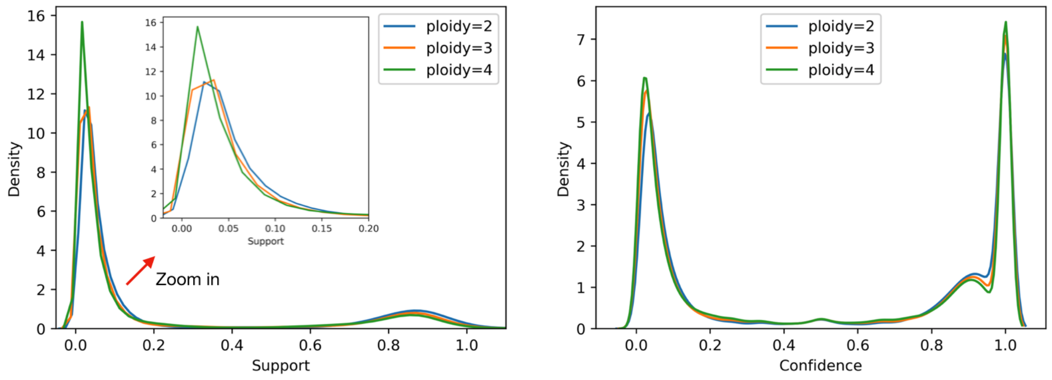
Density
Support
Zoom in
ploidy=2
ploidy=3
ploidy=4
Confidence
ploidy=2
ploidy=3
ploidy=4

(b)

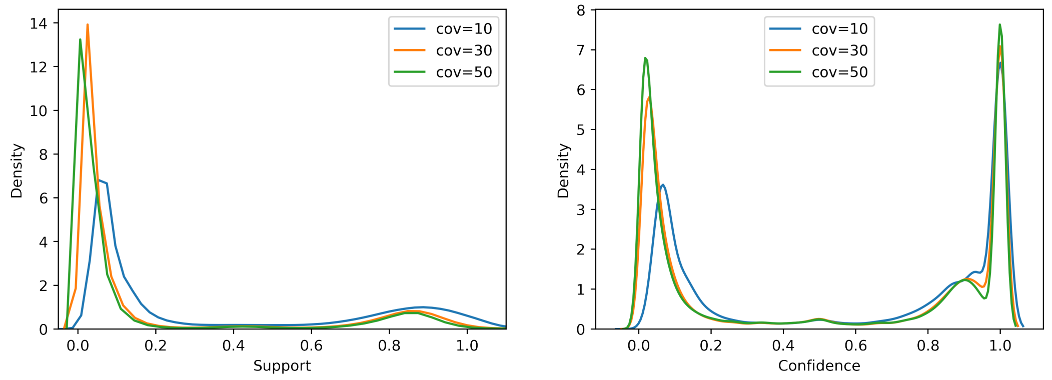
Density
Support
cov=10
cov=30
cov=50
Confidence
cov=10
cov=30
cov=50

(c)

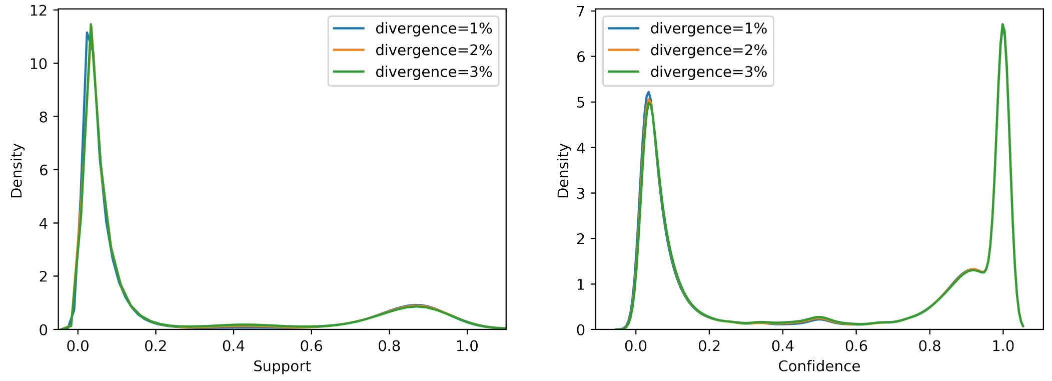
Density
Support
divergence=1%
divergence=2%
divergence=3%
Confidence
divergence=1%
divergence=2%
divergence=3%

(d)

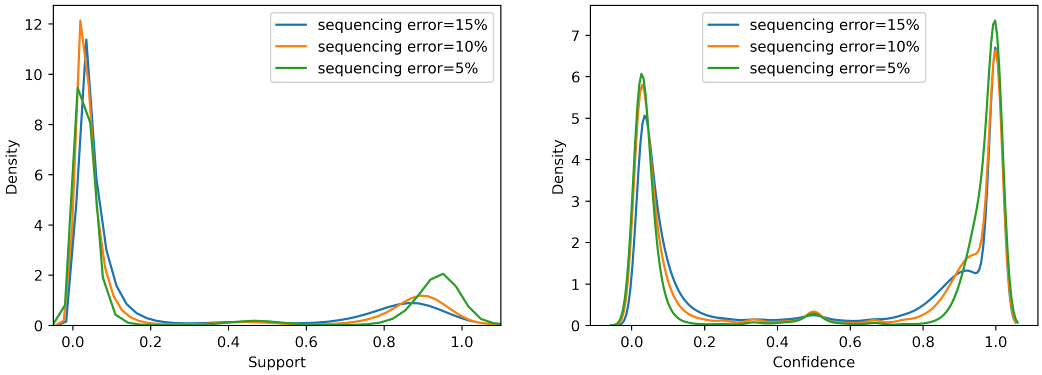

(e)

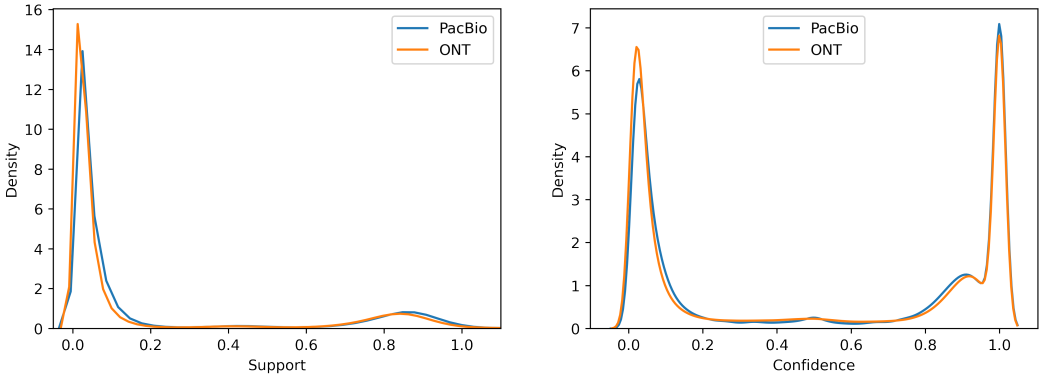

Supplementary Figure 1. Distributions for *Support* and *Confidence* in the raw variation graph in various settings. Note that the *Support* value at the horizontal axis is scaled by the average sequencing coverage. **(a), (b), (c), (d), (e)** show the different settings for ploidy, sequencing coverage, genome divergence, sequencing error rate and sequencing platform, respectively. The original genome sequence (length=50Kbp) used in this experiment is generated by randomly choosing $\{A, T, C, G\}$, and the mutated haplotypes are generated by introducing random mutations artificially. Raw data of supporting this figure are provided in the Raw data file.

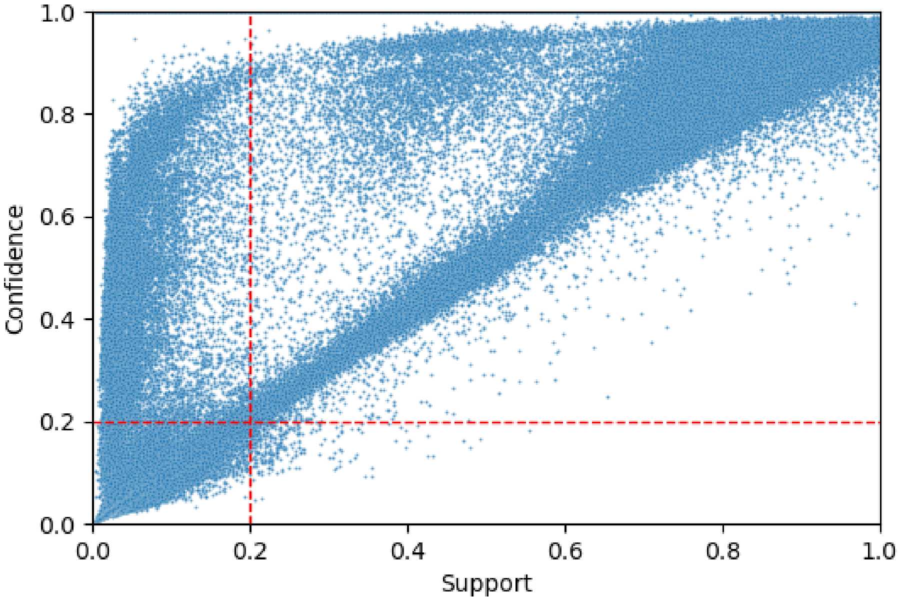

Supplementary Figure 2. *Support* and *Confidence* in the raw variation graph. Note that the *Support* value at the horizontal axis is scaled by the average sequencing coverage. The dataset is from a simulated diploid genome with 2% genome divergence and 50Kbp genome length. The genome sequence is generated randomly and sequencing coverage 20x per haplotype. The reads are simulated PacBio CLR reads with error rate 15%. Each point in the figure represents an edge. The red dashed lines indicate the thresholds of *Support* (scaled) and *Confidence* that used in our experiments. Only the points (edges) on the top right region are retained after graph pruning process. Raw data of supporting this figure are provided in the Raw data file.

Supplementary Methods

Commands and versions of tools used for comparison

- PBSIM2
  `pbsim2 --accuracy-mean 0.9 --hmm_model P6C4.model $ref` #PacBio
  `pbsim2 --accuracy-mean 0.9 --hmm_model R103.model $ref --difference-ratio 23:31:46` #ONT
- Canu v2.1.1
  `canu genomeSize=$genomesize -pacbio $raw_read`
  `canu genomeSize=$genomesize -nanopore $raw_read`
  `canu genomeSize=$genomesize -pacbio-hifi $corrected_read` #run HiFi mode on corrected reads
- Flye v2.8.2-b1689
  `flye --pacbio-raw  $read` #(PacBio)
  `flye --nano-raw  $read` #(ONT)
  `flye --meta --pacbio-raw  $read` #(metagenome assembly)
- Racon v1.4.13
  `racon -f  $raw_read  $overlap  $raw_read >$corrected_read`
- Daccord v0.0.18
  `fasta2DAM reads.dam reads`
  `DBsplit -s256 -x1000 reads.dam`
  `HPC.daligner reads.dam -T$threads| bash`
  `daccord reads.las reads.dam >$corrected_read`
- CONSENT v2.2.2
  `CONSENT-correct --in $raw_read --out $corrected_read  --type PB/ONT`
- QUAST v5.1.0rc1
  `quast.py -r $ref  --min-contig 500 -o out --ambiguity-usage one --fast $fa`
- VeChat v1.1.0
  #(simulated data)
  `vechat -o $corrected_read --platform pb/ont  $raw_read`

  #(simulated data, metagenomic dataset of high complexity)
  `vechat -o $corrected_read --platform pb/ont  --split --split-size 100000 $raw_read`

  # (real data: Yeast pseudo-diploid/Microbial 10-plex metagenome)
  `vechat -o $corrected_read --platform pb --min-identity-cns 0.98 --scrub $raw_read`

  # (real data: NWC)
  `vechat -o $corrected_read --platform pb --min-identity-cns 0.98`
  `--split --split-size 70000 $raw_read`
- VeChat + HiCanu
  `canu genomeSize=$genomesize  -pacbio-hifi $corrected_read`
- VeChat + Canu
  `canu genomeSize=$genomesize  -corrected -pacbio $corrected_read`
- VeChat + Flye
  `flye  --pacbio-corr  $corrected_read`
  `flye --meta  --pacbio-corr  $corrected_read` #(metagenome)
- minimap2 v2.17
- fpa v0.5

- yacrd v0.6.2
- art_illumina v2.3.7
  ```
  art_illumina -sam -i $ref -p -l 150 -ss HS25 -f $depth  -m 200 -s 10 -o $prefix
  ```
- BCALM2
  ```
  bcalm -in  reads.illumina.fq  -kmer-size 31  -abundance-min 10 -nb-cores 32 -out bcalm.out
  ```
- GraphAligner v1.0.16
  ```
  GraphAligner -g $dbg -f $tgs_reads  -a out.gaf -t 48 -x dbg --corrected-out corrected.fa
  ```
- fastp v0.20.1
  ```
  fastp -i $raw_reads -o out.qc.fq --length_required 1000 -q 10 --cut_front --cut_tail
  --cut_window_size  4 --cut_mean_quality 10 --thread 16 --trim_front1 20
  ```

References

[1] Francesca Di Giallonardo, Armin Töpfer, Melanie Rey, Sandhya Prabhakaran, Yannick Duport, Christine Leemann, Stefan Schmutz, Nottania K Campbell, Beda Joos, Maria Rita Lecca, et al. Full-length haplotype reconstruction to infer the structure of heterogeneous virus populations. *Nucleic acids research*, 42(14):e115–e115, 2014.

[2] Jasmijn A Baaijens, Amal Zine El Aabidine, Eric Rivals, and Alexander Schönhuth. De novo assembly of viral quasispecies using overlap graphs. *Genome research*, 27(5):835–848, 2017.

[3] Jasmijn A Baaijens, Bastiaan Van der Roest, Johannes Köster, Leen Stougie, and Alexander Schönhuth. Full-length de novo viral quasispecies assembly through variation graph construction. *Bioinformatics*, 35(24):5086–5094, 2019.

[4] Xiao Luo, Xiongbin Kang, and Alexander Schönhuth. Strainline: full-length de novo viral haplotype reconstruction from noisy long reads. *Genome biology*, 23(1):1–27, 2022.
