# Supplementary material for: VeChat: correcting errors in long reads using variation graphs
Source: Nat Commun. 2022 Nov 4;13:6657. doi: 10.1038/s41467-022-34381-8 (PMC9636371; doi:10.1038/s41467-022-34381-8)
Supplement: Supplementary file 3 — Description of Additional Supplementary Files [file 41467_2022_34381_MOESM3_ESM.pdf]

Description of Additional Supplementary Files

We provided a Supplementary File: “Supplementary Data 1-3.xlsx”, with three tabs:

File Name: Supplementary Data 1
Description: “Genome descriptions of simulated sequencing data sets”.

File Name: Supplementary Data 2
Description: “Average sequencing coverage information of simulated metagenome data sets”.

File Name: Supplementary Data 3
Description: “Genome descriptions of real sequencing data sets”.
